# (*E*)-4-Chloro-*N*′-(2-chloro­benzyl­idene)benzohydrazide

**DOI:** 10.1107/S1600536809035739

**Published:** 2009-09-09

**Authors:** Guo-Biao Cao

**Affiliations:** a Department of Chemistry, Ankang University, Ankang Shanxi 725000, People’s Republic of China

## Abstract

The title compound, C_14_H_10_Cl_2_N_2_O, was synthesized by the reaction of 2-chloro­benzaldehyde with an equimolar quantity of 4-chloro­benzohydrazide in methanol. The mol­ecule displays an *E* configuration about the C=N bond. The dihedral angle between the two benzene rings is 8.6 (2)°. In the crystal structure, mol­ecules are linked through inter­molecular N—H⋯O hydrogen bonds, forming chains running along the *c* axis.

## Related literature

For examples of the crystal structures of hydrazone compounds, see: Mohd Lair *et al.* (2009 ▶); Fun *et al.* (2008 ▶); Li & Ban (2009 ▶); Zhu *et al.* (2009 ▶); Yang (2007 ▶); You *et al.* (2008 ▶). For the hydrazone compounds previously reported by our group, see: Qu *et al.* (2008 ▶); Yang *et al.* (2008 ▶), Cao & Lu (2009*a* ▶,*b* ▶), Cao (2009*a* ▶,*b* ▶).
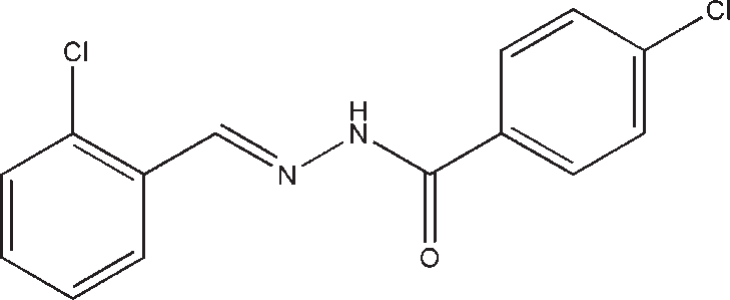


## Experimental

### 

#### Crystal data

C_14_H_10_Cl_2_N_2_O
*M* *_r_* = 293.14
Monoclinic, 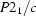

*a* = 10.9140 (4) Å
*b* = 13.3253 (4) Å
*c* = 9.1283 (3) Å
β = 96.165 (2)°
*V* = 1319.87 (8) Å^3^
*Z* = 4
Mo *K*α radiation
μ = 0.48 mm^−1^
*T* = 298 K
0.20 × 0.20 × 0.18 mm

#### Data collection

Bruker SMART 1K diffractometer
Absorption correction: multi-scan (*SADABS*; Bruker, 2001 ▶) *T*_min_ = 0.910, *T*_max_ = 0.918
7970 measured reflections
2863 independent reflections
2099 reflections with *I* > 2σ(*I*)
*R*_int_ = 0.023

#### Refinement

*R*[*F* ^2^ > 2σ(*F* ^2^)] = 0.037
*wR*(*F* ^2^) = 0.103
*S* = 1.05
2863 reflections
176 parameters
1 restraint
H atoms treated by a mixture of independent and constrained refinement
Δρ_max_ = 0.18 e Å^−3^
Δρ_min_ = −0.23 e Å^−3^

### 

Data collection: *SMART* (Bruker, 2007 ▶); cell refinement: *SAINT* (Bruker, 2007 ▶); data reduction: *SAINT*; program(s) used to solve structure: *SHELXTL* (Sheldrick, 2008 ▶); program(s) used to refine structure: *SHELXTL*; molecular graphics: *SHELXTL*; software used to prepare material for publication: *SHELXTL*.

## Supplementary Material

Crystal structure: contains datablocks global, I. DOI: 10.1107/S1600536809035739/om2272sup1.cif

Structure factors: contains datablocks I. DOI: 10.1107/S1600536809035739/om2272Isup2.hkl

Additional supplementary materials:  crystallographic information; 3D view; checkCIF report

## Figures and Tables

**Table 1:** Hydrogen-bond geometry (Å, °)

*D*—H⋯*A*	*D*—H	H⋯*A*	*D*⋯*A*	*D*—H⋯*A*
N2—H2⋯O1^i^	0.889 (9)	2.065 (11)	2.9157 (18)	159.8 (18)

Symmetry code: (i) 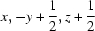.

**Fig. 1.**
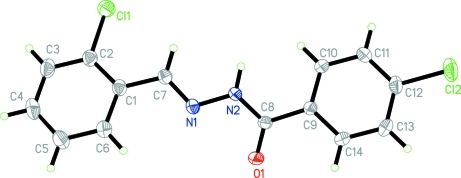
The molecular structure of the title compound with ellipsoids drawn at the 30% probability level.

**Fig. 2.**
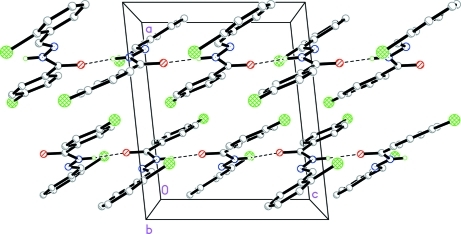
The molecular packing of the title compound, viewed along the b axis. Hydrogen bonds are drawn as dashed lines.

## supplementary crystallographic information

### Comment

In the last few years, the crystal structures of a large number of hydrazone compounds have been reported (Mohd Lair *et al.*, 2009; Fun *et al.*, 2008; Li & Ban, 2009; Zhu *et al.*, 2009; Yang, 2007; You *et al.*, 2008). As a continuation of our work in this area (Qu *et al.*, 2008; Yang *et al.*, 2008; Cao & Lu, 2009a,b; Cao, 2009a,b), the title new hydrazone compound, derived from the reaction of 2-chlorobenzaldehyde with an equimolar quantity of 4-chlorobenzohydrazide, is reported.

In the title compound, Fig. 1, the dihedral angle between the two benzene rings is 8.6 (2)°. The molecule displays an *E* configuration about the C═N bond. In the crystal structure, molecules are linked through intermolecular N—H···O hydrogen bonds, Table 1, to form chains running along the *c* axis, Fig. 2.

### Experimental

The compound was prepared by refluxing equimolar quantities of 2-chlorobenzaldehyde with 4-chlorobenzohydrazide in methanol. Colorless block-like crystals were formed by slow evaporation of the solution in air.

### Refinement

H2 was located in a difference Fourier map and refined isotropically, with the N—H distance restrained to 0.90 (1) Å. The other H atoms were placed in idealized positions and constrained to ride on their parent atoms, with C—H distances of 0.93 Å, and with *U*_iso_(H) set at 1.2*U*_eq_(C).

### Crystal data

**Table:**

C_14_H_10_Cl_2_N_2_O	*F*(000) = 600
*M**_r_* = 293.14	*D*_x_ = 1.475 Mg m^−^^3^
Monoclinic, *P*2_1_/*c*	Mo *K*α radiation, λ = 0.71073 Å
Hall symbol: -P 2ybc	Cell parameters from 2259 reflections
*a* = 10.9140 (4) Å	θ = 2.4–26.5°
*b* = 13.3253 (4) Å	µ = 0.48 mm^−^^1^
*c* = 9.1283 (3) Å	*T* = 298 K
β = 96.165 (2)°	Block, colorless
*V* = 1319.87 (8) Å^3^	0.20 × 0.20 × 0.18 mm
*Z* = 4	

### Data collection

**Table:**

Bruker SMART 1K diffractometer	2863 independent reflections
Radiation source: fine-focus sealed tube	2099 reflections with *I* > 2σ(*I*)
graphite	*R*_int_ = 0.023
ω scans	θ_max_ = 27.0°, θ_min_ = 1.9°
Absorption correction: multi-scan (*SADABS*; Bruker, 2001)	*h* = −10→13
*T*_min_ = 0.910, *T*_max_ = 0.918	*k* = −17→16
7970 measured reflections	*l* = −11→11

### Refinement

**Table:**

Refinement on *F*^2^	Primary atom site location: structure-invariant direct methods
Least-squares matrix: full	Secondary atom site location: difference Fourier map
*R*[*F*^2^ > 2σ(*F*^2^)] = 0.037	Hydrogen site location: inferred from neighbouring sites
*wR*(*F*^2^) = 0.103	H atoms treated by a mixture of independent and constrained refinement
*S* = 1.05	*w* = 1/[σ^2^(*F*_o_^2^) + (0.0443*P*)^2^ + 0.3326*P*] where *P* = (*F*_o_^2^ + 2*F*_c_^2^)/3
2863 reflections	(Δ/σ)_max_ = 0.001
176 parameters	Δρ_max_ = 0.18 e Å^−^^3^
1 restraint	Δρ_min_ = −0.23 e Å^−^^3^

### Special details

**Table:**

Geometry. All esds (except the esd in the dihedral angle between two l.s. planes) are estimated using the full covariance matrix. The cell esds are taken into account individually in the estimation of esds in distances, angles and torsion angles; correlations between esds in cell parameters are only used when they are defined by crystal symmetry. An approximate (isotropic) treatment of cell esds is used for estimating esds involving l.s. planes.
Refinement. Refinement of F^2^ against ALL reflections. The weighted R-factor wR and goodness of fit S are based on F^2^, conventional R-factors R are based on F, with F set to zero for negative F^2^. The threshold expression of F^2^ > 2sigma(F^2^) is used only for calculating R-factors(gt) etc. and is not relevant to the choice of reflections for refinement. R-factors based on F^2^ are statistically about twice as large as those based on F, and R- factors based on ALL data will be even larger.

### Fractional atomic coordinates and isotropic or equivalent isotropic displacement parameters (Å^2^)

**Table:**

	*x*	*y*	*z*	*U*_iso_*/*U*_eq_	
Cl1	0.23751 (6)	0.62666 (4)	1.13145 (6)	0.0669 (2)	
Cl2	0.45481 (6)	−0.17289 (4)	1.34138 (7)	0.0725 (2)	
N1	0.19376 (14)	0.33512 (10)	0.93524 (16)	0.0400 (4)	
N2	0.23897 (15)	0.25647 (11)	1.02386 (16)	0.0394 (4)	
O1	0.27146 (14)	0.16448 (10)	0.82336 (13)	0.0503 (4)	
C1	0.13324 (16)	0.50527 (13)	0.9130 (2)	0.0404 (4)	
C2	0.15065 (18)	0.60340 (14)	0.9639 (2)	0.0442 (4)	
C3	0.1036 (2)	0.68464 (15)	0.8830 (2)	0.0543 (5)	
H3	0.1166	0.7494	0.9193	0.065*	
C4	0.0371 (2)	0.66901 (17)	0.7480 (3)	0.0600 (6)	
H4	0.0048	0.7234	0.6929	0.072*	
C5	0.0185 (2)	0.57327 (17)	0.6945 (2)	0.0586 (6)	
H5	−0.0263	0.5630	0.6032	0.070*	
C6	0.06591 (18)	0.49246 (15)	0.7758 (2)	0.0487 (5)	
H6	0.0527	0.4280	0.7383	0.058*	
C7	0.18227 (18)	0.41876 (13)	0.9994 (2)	0.0423 (4)	
H7	0.2048	0.4250	1.1002	0.051*	
C8	0.27714 (17)	0.17307 (13)	0.95759 (19)	0.0376 (4)	
C9	0.32468 (17)	0.08966 (12)	1.05677 (18)	0.0359 (4)	
C10	0.37559 (18)	0.10432 (13)	1.20078 (19)	0.0411 (4)	
H10	0.3812	0.1688	1.2399	0.049*	
C11	0.41803 (19)	0.02376 (14)	1.2864 (2)	0.0477 (5)	
H11	0.4537	0.0340	1.3824	0.057*	
C12	0.40759 (18)	−0.07139 (14)	1.2298 (2)	0.0456 (5)	
C13	0.3595 (2)	−0.08810 (14)	1.0865 (2)	0.0570 (6)	
H13	0.3541	−0.1529	1.0484	0.068*	
C14	0.3195 (2)	−0.00707 (14)	1.0001 (2)	0.0521 (5)	
H14	0.2885	−0.0174	0.9023	0.063*	
H2	0.2458 (17)	0.2649 (15)	1.1210 (11)	0.048 (6)*	

### Atomic displacement parameters (Å^2^)

**Table:**

	*U*^11^	*U*^22^	*U*^33^	*U*^12^	*U*^13^	*U*^23^
Cl1	0.0997 (5)	0.0446 (3)	0.0528 (3)	−0.0018 (3)	−0.0077 (3)	−0.0010 (2)
Cl2	0.0851 (5)	0.0532 (3)	0.0794 (4)	0.0270 (3)	0.0106 (3)	0.0243 (3)
N1	0.0515 (9)	0.0326 (8)	0.0350 (8)	0.0001 (6)	0.0009 (7)	0.0062 (6)
N2	0.0597 (10)	0.0302 (7)	0.0272 (7)	0.0026 (7)	−0.0002 (7)	0.0020 (6)
O1	0.0832 (10)	0.0410 (7)	0.0264 (6)	−0.0001 (6)	0.0039 (6)	−0.0008 (5)
C1	0.0451 (11)	0.0377 (9)	0.0397 (10)	0.0033 (8)	0.0101 (8)	0.0068 (8)
C2	0.0504 (11)	0.0396 (10)	0.0434 (10)	0.0023 (8)	0.0084 (8)	0.0055 (8)
C3	0.0655 (14)	0.0364 (10)	0.0616 (13)	0.0059 (9)	0.0098 (11)	0.0081 (9)
C4	0.0627 (14)	0.0519 (13)	0.0652 (14)	0.0149 (10)	0.0053 (11)	0.0224 (11)
C5	0.0607 (14)	0.0623 (14)	0.0506 (12)	0.0105 (11)	−0.0043 (10)	0.0106 (10)
C6	0.0542 (12)	0.0436 (11)	0.0478 (11)	0.0055 (9)	0.0029 (9)	0.0033 (9)
C7	0.0573 (12)	0.0348 (9)	0.0347 (9)	0.0020 (8)	0.0049 (8)	0.0045 (7)
C8	0.0491 (11)	0.0331 (9)	0.0301 (9)	−0.0065 (7)	0.0014 (8)	0.0004 (7)
C9	0.0449 (10)	0.0305 (8)	0.0328 (9)	−0.0012 (7)	0.0057 (7)	−0.0004 (7)
C10	0.0544 (12)	0.0331 (9)	0.0354 (10)	−0.0001 (8)	0.0023 (8)	−0.0028 (7)
C11	0.0595 (13)	0.0460 (11)	0.0363 (10)	0.0074 (9)	−0.0007 (9)	0.0030 (8)
C12	0.0494 (11)	0.0381 (10)	0.0500 (11)	0.0111 (8)	0.0088 (9)	0.0102 (8)
C13	0.0808 (16)	0.0299 (9)	0.0596 (13)	0.0065 (10)	0.0046 (11)	−0.0057 (9)
C14	0.0780 (15)	0.0385 (10)	0.0379 (11)	0.0037 (10)	−0.0026 (9)	−0.0079 (8)

### Geometric parameters (Å, °)

**Table:**

Cl1—C2	1.739 (2)	C5—C6	1.377 (3)
Cl2—C12	1.7379 (19)	C5—H5	0.9300
N1—C7	1.272 (2)	C6—H6	0.9300
N1—N2	1.3822 (19)	C7—H7	0.9300
N2—C8	1.352 (2)	C8—C9	1.491 (2)
N2—H2	0.889 (9)	C9—C10	1.385 (2)
O1—C8	1.225 (2)	C9—C14	1.388 (2)
C1—C6	1.393 (3)	C10—C11	1.378 (2)
C1—C2	1.394 (3)	C10—H10	0.9300
C1—C7	1.465 (2)	C11—C12	1.369 (3)
C2—C3	1.378 (3)	C11—H11	0.9300
C3—C4	1.377 (3)	C12—C13	1.374 (3)
C3—H3	0.9300	C13—C14	1.380 (3)
C4—C5	1.373 (3)	C13—H13	0.9300
C4—H4	0.9300	C14—H14	0.9300
			
C7—N1—N2	116.21 (14)	N1—C7—H7	120.3
C8—N2—N1	117.98 (14)	C1—C7—H7	120.3
C8—N2—H2	123.6 (13)	O1—C8—N2	122.62 (16)
N1—N2—H2	118.3 (13)	O1—C8—C9	120.95 (16)
C6—C1—C2	117.03 (17)	N2—C8—C9	116.41 (14)
C6—C1—C7	120.97 (17)	C10—C9—C14	118.77 (16)
C2—C1—C7	122.00 (17)	C10—C9—C8	123.30 (15)
C3—C2—C1	121.92 (19)	C14—C9—C8	117.92 (15)
C3—C2—Cl1	117.90 (16)	C11—C10—C9	120.28 (17)
C1—C2—Cl1	120.15 (14)	C11—C10—H10	119.9
C4—C3—C2	119.4 (2)	C9—C10—H10	119.9
C4—C3—H3	120.3	C12—C11—C10	119.86 (17)
C2—C3—H3	120.3	C12—C11—H11	120.1
C5—C4—C3	120.14 (19)	C10—C11—H11	120.1
C5—C4—H4	119.9	C11—C12—C13	121.14 (17)
C3—C4—H4	119.9	C11—C12—Cl2	119.47 (15)
C4—C5—C6	120.2 (2)	C13—C12—Cl2	119.39 (15)
C4—C5—H5	119.9	C12—C13—C14	118.83 (18)
C6—C5—H5	119.9	C12—C13—H13	120.6
C5—C6—C1	121.34 (19)	C14—C13—H13	120.6
C5—C6—H6	119.3	C13—C14—C9	121.04 (18)
C1—C6—H6	119.3	C13—C14—H14	119.5
N1—C7—C1	119.33 (16)	C9—C14—H14	119.5

### Hydrogen-bond geometry (Å, °)

**Table:**

*D*—H···*A*	*D*—H	H···*A*	*D*···*A*	*D*—H···*A*
N2—H2···O1^i^	0.89 (1)	2.07 (1)	2.9157 (18)	160 (2)

Symmetry codes: (i) *x*, −*y*+1/2, *z*+1/2.
